# Remote *p*-type Doping in GaSb/InAs Core-shell Nanowires

**DOI:** 10.1038/srep10813

**Published:** 2015-06-01

**Authors:** Feng Ning, Li-Ming Tang, Yong Zhang, Ke-Qiu Chen

**Affiliations:** 1 Department of Applied Physics, School of Physics and Electronics, Hunan University, Changsha 410082, China

## Abstract

By performing first-principles calculation, we investigated the electronic properties of remotely p-type doping GaSb nanowire by a Zn-doped InAs shell. The results show that for bare zinc-blende (ZB) [111] GaSb/InAs core-shell nanowire the Zn *p*-type doped InAs shell donates free holes to the non-doped GaSb core nanowire without activation energy, significantly increasing the hole density and mobility of nanowire. For Zn doping in bare ZB [110] GaSb/InAs core-shell nanowire the hole states are compensated by surface states. We also studied the behaviors of remote *p*-type doing in two-dimensional (2D) GaSb/InAs heterogeneous slabs, and confirmed that the orientation of nanowire side facet is a key factor for achieving high efficient remote *p*-type doping.

Semiconductor nanowires (NWs) have attracted considerable attention due to their potential applications as field-effect transistors (FETs)^1^,^2^, miniaturized optic-electronic devices^3^,^4^, phonon devices^5^,^6^,^7^,^8^, and heat cable^9^. GaSb is a III-V semiconductor, and has high hole mobility (850–10800 cm^2^/Vs), low carriers effective masses, and small direct band gap among the semiconductors^10^. Recently, *p*-type GaSb-based III-V semiconductors NWs have been a subject of intensive research owning to their excellent electronic properties. Nevertheless, the electronic properties of GaSb nanowires are much different from that of bulk phase^11^,^12^. For instance, the band gaps and carriers effective masses increase with the decrease in diameters^12^, and surface roughness scattering causes a obvious decrease in current and carriers mobilities of GaSb NWs^13^,^14^. Yang *et al.*^15^ found that the hole mobility of GaSb NW in 180 nm diameter is only 30 cm^2^/Vs which is nearly 30 times lower than that of bulk GaSb. A serious obstacle for GaSb NW in practical application is presented.

Most recently, a novel method that impurity-doing in the shell has been proposed to increase the hole densities and mobilities in core-shell NWs^16^,^17^,^18^,^19^, where the impurity atoms are in the shell that donates carriers to the non-doped core nanowire, i.e., to remotely dope nanowires. The free electrons (holes) spontaneously fall into the energy well of the non-doped core NW because of a conduction (valence) band offset at core/shell interface. Dillen *et al.*^17^ experimentally incorporated a B-doped layer into the shell of Ge/Si core-shell NWs. They found that the mobility of Ge/Si core-shell NW with B-doped in the shell is 700-1800 cm^2^/Vs, whereas the non-doped core-shell NW shows a mobility of 100–280 cm^2^/Vs. However, considering the successful fabrication of GaSb/InAs core-shell NWs^20^, few effort has been devoted to increasing the hole mobilities of GaSb/InAs core-shell NWs using remote doping method. Realizing remote *p*-type doing in bare core-shell NWs is considered complicated, since the efficiency of remote *p*-type doping highly depends on the surface states, side facets, and valence band offset of core-shell NWs^21^,^22^,^23^. As we know, the surface atoms of NWs are not saturated by foreign atoms due to the high-vacuum environment during the growth^21^. High surface-to-volume ratio of nanowires leads to strong dependence of their electrical properties on surface states and side facets of NWs^21^,^22^. The surface donors states can easily compensate the external acceptors states. Additionally, the remote *p*-type doing in core-shell NW highly depends on the type-II valence band offset which is dependent on the NW diameter and core/shell composition ration^23^. A systematic theoretical work is required to explore the critical factor for realizing remote doping in bare GaSb/InAs core-shell NWs.

In the present work, we study the Zn remote doping in bare zinc-blende (ZB) [111] and [110] GaSb/InAs core-shell NWs with different side facets. The effect of side facets on *p*-type doping was considered. The results show that for bare ZB [111] GaSb/InAs core-shell NW, *p*-type Zn-doped InAs-shell donates one-dimensional hole gas to the non-doped GaSb core NW without thermal activation energy, increasing hole densities with high mobilities. In contrast, the acceptors states in the bare [110] GaSb/InAs core-shell NWs are compensated by the surface states. To further understand the effects of side facets of NWs on remotely doping in core-shell NWs, we explored the behavior of Zn *p*-type remote doing in two-dimensional GaSb/InAs heterogeneous slabs. The results demonstrated that the orientation of nanowire side fact determine the efficiency of *p*-type doping. The GaSb/InAs core-shell NWs that with the lateral surface where are In-As atomic pairs termination and As atoms termination are favor for effective remote *p*-type doping.

## Results

**Figure 1:**
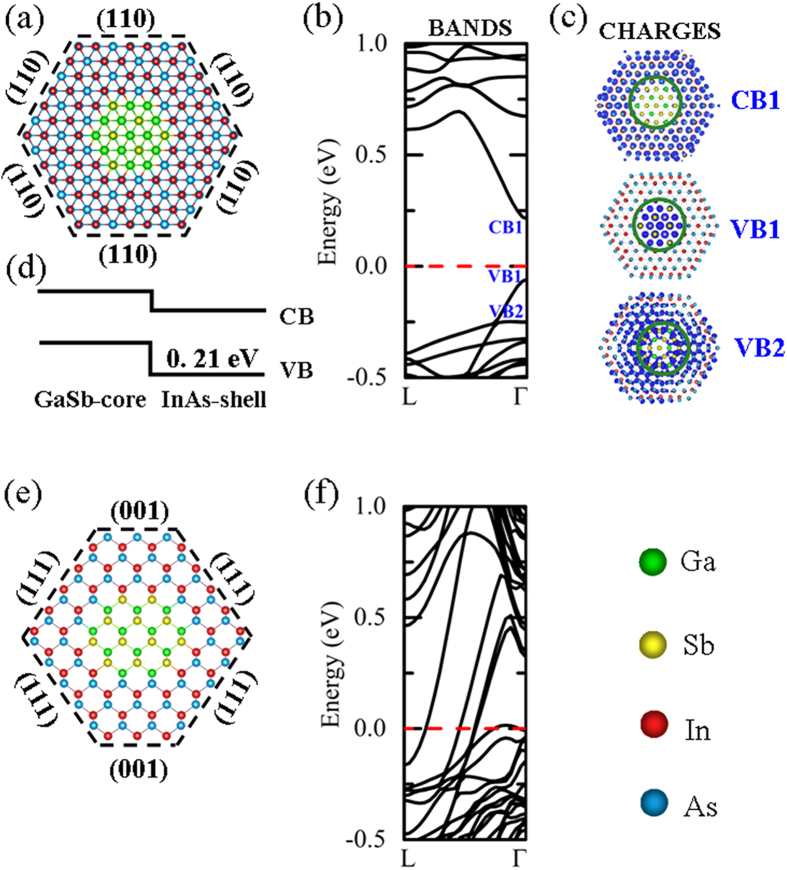
(**a**) Cross-section viewed of bare ZB [111] GaSb-core/InAs-shell nanowire structure with six (110) side facets terminated with In-As atomic pairs, and with a diameter about *D* = 3 nm and a core diameter *D*_*C*_ = 1 nm, (**b**) the corresponding band structure (BANDS), (**c**) charge density analysis of specific states (CHARGES), and (**d**) calculated band alignment (band gap not in scale). CB and VB represent conduction band and valence band, respectively. (**e**) Cross-section viewed of ZB [110] GaSb-core/InAs-shell nanowire with four (111) side facets and two (100) side facets where are either As atoms termination or In atoms terminations, and with a diameter about *D* = 3 nm and a core diameter *D*_*C*_ = 1.3 nm, and (**f**) the corresponding band structure. Green, yellow, red, and blue balls correspond to Ga, Sb, In, and As atoms, respectively.

We constructed the bare ZB GaSb/InAs core-shell NW along the [111] and [110] directions as shown in Fig. 1(a), respectively. The optimized lattice constants of the zinc-blende (ZB) GaSb (InAs) bulk materials a = 6.210 (6.184) Å, similar to experimental and theoretical values^24^,^25^. Consequently, the ZB GaSb/InAs heterostructure has a negligible interface strain owning to the small lattice mismatch (0.4%). All the core-shell NWs are assumed to be infinitely long with a periodic length 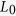, oriented along the *z*-direction. For instance, the length 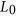 of periodic unit cell of ZB [111] GaSb NW is 10.757 Å and that of ZB [111] InAs NW is 10.712 Å, the lattice mismatch between the GaSb-core and InAs-shell is only 0.42%. Then, we extracted the average lattice constants of GaSb and InAs NW and optimized the GaSb/InAs lattice constant to further release the interface strain. In Fig. 1(b), the band structure of bare ZB [111] GaSb/InAs core-shell NW is presented. It shows a typical semiconductor band characteristic without surface states within the band gap. For the ZB [111] core-shell NWs with (110) side facets where are In-As atomic pairs terminations, the surface states are found in the conduction and valence bands continua, respectively, owning to the reconstruction of (110) surfaces atoms after relaxation^26^. The conduction band minimum (CBM), valence band maximum (VBM), and the second valence band are labeled by CB1, VB1, and VB2, respectively, and their charge density distributions are plotted out in Fig. 1(c). Clearly, it is found that the charge density of CB1 state mainly localizes in InAs shell and that of VB1 state localizes in GaSb core. A quasi type-II band alignment is indicated. The charge distribution of VB2 state is spread throughout the core-shell NW cross-section. A valence band offset (VBO) between the GaSb core and InAs shell is suggested. Based on the operational band offset estimated approach introduced by Yang *et al.*^23^, the energy difference of 0.21 eV between VB1 and VB2 was taken as the operational valence band offset. A band alignment schematic was obtained, as shown in Fig. 1(d). However, for bare ZB [110] GaSb/InAs core-shell NW, its band structure displays a metallic feature as seen in Fig. 1(f). The surface states originating from the unsaturated dangling bonds locate in the band gap. It means that the external impurity acceptors states could be compensated by the surface free electrons. Thus, the electronic properties of *p*-type doping in bare ZB [111] GaSb/InAs core-shell NW are focused on in our following discussions.

**Figure 2:**
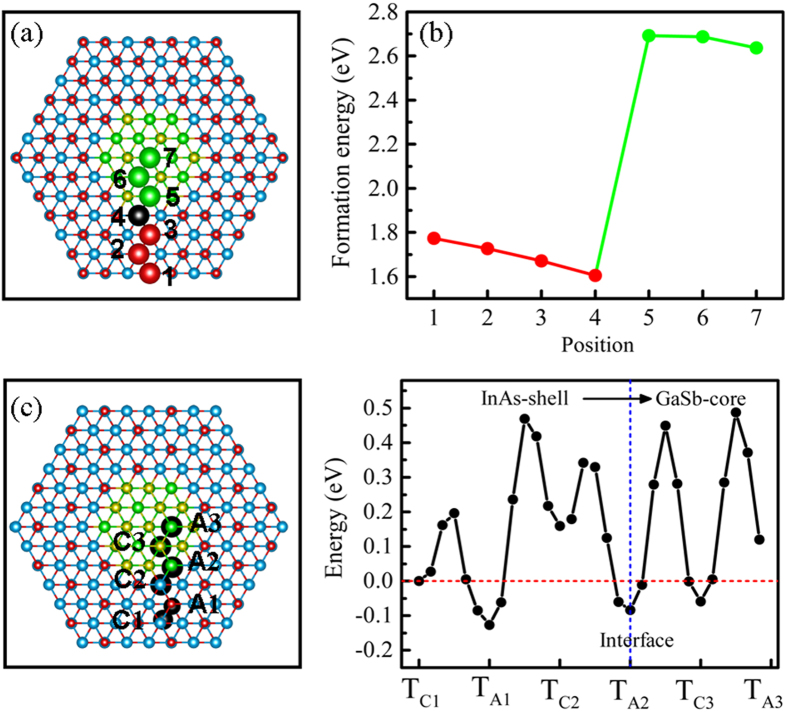
(**a**) Cross-section viewed of bare ZB [111] GaSb/InAs core-shell nanowire, the big balls represent the cations atoms positions of Zn substituting, and the back ball corresponds to Zn atom at the most stable substituting position, (**b**) Formation energy as a function of Zn substituting positions at neutral charge state. (**c**) The diffusion pathways of Zn interstitial defect in core-shell nanowires starting from the T_C1_ site, (**d**) the diffusion barriers along the pathways. T_C_ and T_A_ are the interstitial tetrahedral sites where the nearest neighbors atoms are cation and anion atoms, respectively. The T_C1_ site is the Zn initial site. Energies are referenced to the system energy of Zn atom in T_C1_ site.

We considered Zn doping in GaSb-core/InAs-shell NWs which can serve as an acceptor by substituting In atoms (Zn_In_) in the shell or Ga atoms (Zn_Ga_) in the core. To examine the Zn preferable substituting cations positions, the formation energies are addressed in Fig. 2(b). It is observed that the formation energies (Δ*H*_*f*_) as a function of substituting positions. For the case of Zn_In_ in the InAs shell, the formation energies slightly decrease with the distance from the lateral surface to the inner of InAs-shell NW. The formation energy is the lowest for Zn substituting the interfacial In-atom in the site 4. However, formation energies significantly increase for Zn_Ga_ in the GaSb core NW, and the formation energies are higher by ~1.0 eV than that of Zn doping in the InAs shell. The discrepancy of formation energies for different doping positions can be attributed to the change in bonds lengths and the chemical environment between the impurity and host atoms. For doping in the InAs shell, it is found that when the Zn substitute the In atoms from lateral surface (site 1 position) to interface (site 4 position), the average bonds lengths between Zn and the nearest neighbor As atoms shrunk by 0.1705 Å, 0.1602 Å, 0.1610 Å, and 0.1622 Å, respectively, due to the obvious atomic radius difference between Zn atom (1.25 Å) and In atoms (1.44 Å). In the nanowires structure, the surface Zn dopant atom only has three coordinated atoms which can fully relax the strain causing the obvious change in surface bonds. As a result, the bonds between the Zn and surface As atoms shrink more obviously than that between the Zn and inner region As atoms, contributing to high energies. Therefore, the formation energies for Zn substituting surface In atom is higher than the case for substituting inner region In atoms. Furthermore, the interface strain slightly turns the interface sites lower in energy. In addition, as the Zn atomic radius (1.25 Å) is similar to the Ga atomic radius (1.26 Å), the change in bonds lengths between Zn and Sb atoms is small, as a result, the formation energies difference (0.2 eV) between Zn substitutes different In atoms sites is a little larger than formation energies difference (0.1 eV) between Zn substitutes different core Ga atoms due to high energetic cost of the Zn-As shrinkable bonds. For the case of Zn occupying the Ga atoms of the GaSb core (Zn_Ga_), the Sb 4p states are more delocalized than As 4p states, resulting in the interaction between the Zn and nearest Sb atoms greater than that between Zn and nearest As atoms, which leans to high formation energies for substituting core Ga atoms. In addition, electronegativity of As atoms is greater than Sb atoms electronegativity. So after Zn substitutes the In and Ga atoms, respectively, the forming bonds between Zn and nearest As atoms are more stable than that between Zn and nearest neighbor Sb atoms, causing the low energy when Zn doping the In atoms in the InAs-shell. Finally, we have investigated the barriers when an interstitial Zn atom diffuses from surface toward to the GaSb-core using the climbing-image nudged-elastic-band method (CI-NEB)^27^. Here, we only considered the interstitial sites diffusion. The diffusion path is a straight line between T_C_ and T_A_ interstitial tetrahedral sites, as shown in Fig. 2(c). T_C_ and T_A_ are the interstitial tetrahedral sites where the nearest neighbors atoms are cation and anion atoms, respectively. The transition saddle point between two tetrahedral positions is found to be at the hexagonal site. From Fig. 2(d), we found that the diffusion barrier decreases when starting from the T_C_ site, but increases when starting from the T_A_, for instance, the diffusion barrier is lower for T_C1_ to T_A1_ sites than that for T_A1_ to T_C2_ sites. The Zn interstitial is more stable in the T_A_ site than in the T_C_ site, similar to the case of Zn interstitial in the II-VI semiconductors^28^,^29^. After overcoming a large barrier crossing surface-to-inner region, it can be inferred that Zn interstitial can reach the interface. The previous experimental observation revealed that Zn insertion in InAs nanowires is layer by layer from surface to inner region by gas-phase surface diffusion with the temperatures available at experimental conditions, and finally Zn atoms can be distributed along the InAs NW radius^30^.

**Figure 3:**
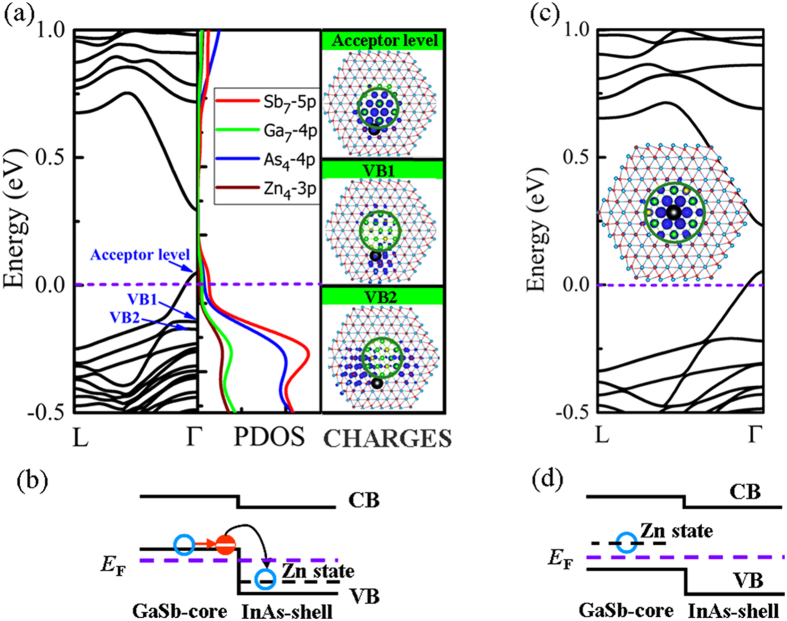
(**a**) Band structures (left panel), projected density of states (PDOS) (middle panel), charge density distributions (CHARGES) (right panel) of acceptor level, VB1, and VB2, respectively, and (**b**) the doping mechanism schematic model for Zn substituting the interfacial In atom (Zn_In_) in the site 4 of ZB [111] GaSb/InAs core-shell NW. (**c**) Band structure for Zn substituting central Ga atoms (Zn_Ga_) in the site 7 of GaSb-core region, the inset represents the charge density distribution of Zn_Ga_ acceptor state, and (**d**) the corresponding doping mechanism schematic model. The dotted lines represent the Fermi level. The isosurface value of charge density is at the 30% of the maximum.

In Fig. 3(a), we give the band structure, projected density of states (PDOS), and charge density analysis of Zn *p*-type doping in the site 4 corresponding the most stable Zn_In_ position. From the band structure, as seen in Fig. 3(a), we clearly see it presents a typical *p*-type characteristic. An acceptor level crosses the Fermi level. The PDOS of Ga-Sb atomic pair in the site 7 of the core and Zn-As pair in the site 4 of the shell was shown in the middle panel of Fig. 3(a). It can be observed that the PDOS near the acceptor state is mainly composed of the Sb 5p states. The charge density distribution of the acceptor level is plotted out in the right panel of Fig. 3(a). We find that the charge distribution of acceptor state is made of GaSb-core state. These results demonstrate that the acceptor state mainly localizes at the non-doped GaSb core atoms. It is different from the previous works that the wave function of acceptor state would highly localize around the dopants atoms^31^. One has to go down below the valence band to find the Zn impurity state labeled by VB1 state. The corresponding charge density distribution is confined at As atoms around the dopant Zn atom. The first InAs-shell level labeled by VB2 state is found below the Zn impurity state, which has a charge distribution mainly localize in the shell region. This doping mechanism originates from the type-II band offset between GaSb core and InAs shell. A doping schematic model of band offset for Zn doping is shown in Fig. 3(b). As seen in Fig. 3(b), the GaSb state stands above the Zn impurity acceptor and InAs shell states. The electrons of GaSb core naturally fall into the Zn impurity hole state without the need of thermal activation (*E*_*a*_ = 0). The Zn impurity hole state is occupied by the electrons, and shifts downward to the valence bands. After electrons transfer, the acceptor state made of GaSb atoms is created above the Fermi level, and one-dimensional hole gas is formed and confined at the non-doped GaSb core atoms. Consequently, the *p*-type doped-InAs shell donates free hole to the non-doped GaSb core, i.e., to remotely dope GaSb core NW. It overcomes the obstacle of high activation and ionization energies of doping in pure GaSb NWs. One should notice that the hole gas in the GaSb core is spatially separated from the ionized Zn impurity. This results in higher carrier mobilities as the strong scattering at ionized dopants in the transport channel is substantially reduced. The carries mobilities of the non-doped and Zn-doped GaSb/InAs core-shell NWs have been estimated based on the deformation potential method proposed by previous works^32^,^33^,^34^,^35^,^36^,^37^, and the calculated results were shown in Table 1. For the case of non-doped GaSb/InAs core-shell NW, the hole mobility (GaSb-dominated) 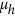 is about 4.48 × 10^3^ cm^2^/Vs, which is obvious larger than the electron mobility (InAs-dominated)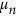 = 2.45 × 10^3^ cm^2^/Vs. The calculated electron mobility in InAs-shell NW is much smaller than that in bulk InAs due to the surface roughness scattering caused by acoustic phonon and surface/interface trap states. This result was similar to the work reported by Ford *et al.*^14^, they found that the 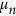 of InAs NWs with a diameter of 7 nm was only 2.5×10^4^ cm^3^/Vs. After Zn doping the shell in GaSb/InAs core-shell NW, the hole mobility 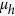 can reach 5.53×10^4^ cm^3^/Vs, which is larger than the hole mobility of non-doped GaSb/InAs core-shell NW. The Zn *p*-type doped InAs-shell contributed holes to the non-doped GaSb-core NW utilizing the valence band offset, and decreased the scattering at ionized dopants and the probability of compensation from the surface state, increasing the hole mobility.

We also studied the electronic properties of Zn substituting Ga atom in the center of GaSb core NW in the site 7 corresponding the most stable Zn_Ga_ position. The band structure and charge density distribution are given in Fig. 3(c). The band structure displays a typical *p*-type band structure feature. The charge density distribution of the acceptor state shown in the inset of Fig. 3(c) reveals that the acceptor state wave function mainly localizes around the Zn impurity. Doping mechanism illustration is schematically sketched in Fig. 3(d). The acceptor state made of Zn atom is higher in energy than GaSb-core and InAs-shell states. The electron of GaSb core cannot be spontaneously transferred into Zn impurity hole state without the help of thermal activation. The band-offset at the GaSb/InAs interface suggests that no enhancement of the doping efficiency in the GaSb core NW.[Table t1]

**Figure 4:**
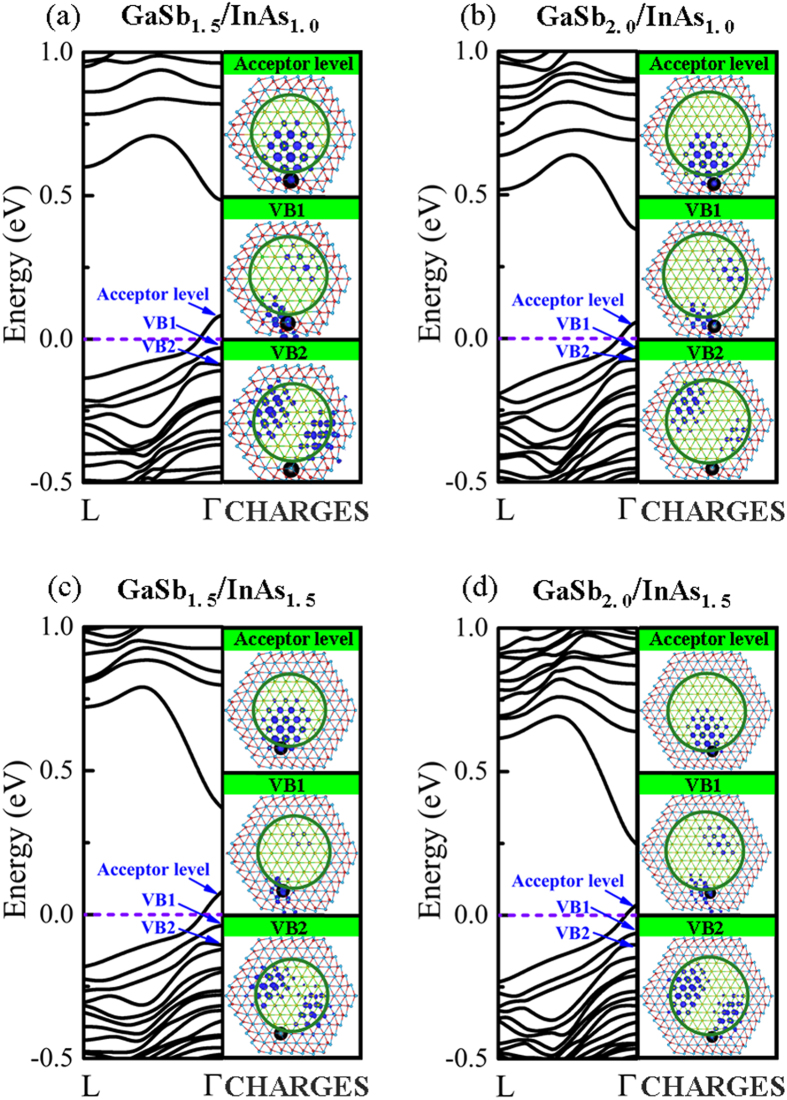
Band structures (left panel) and charge density distributions (CHARGES) (right panel) of acceptor level, VB1, and VB2, respectively, of Zn shell-doping in (**a**) ZB [111] GaSb_1.5_/InAs_1.0_, (**b**) GaSb_2.0_/InAs_1.0_, (**c**) GaSb_1.5_/InAs_1.5_, and (**d**) GaSb_2.0_/InAs_1.5_ core-shell NWs. The index values of GaSb and InAs represent the GaSb-core diameter and the InAs-shell thickness, respectively. The diameters of GaSb-core NW increase from 1.5 to 2.0 nm, and the thicknesses of InAs-shell NW increase from 1.0 to 1.5 nm. The isosurface value of charge density is at the 30% of the maximum. The Zn atom (back ball) substitutes the interface In atom, namely, the energy stable doping-site.

We investigated the electronic properties of Zn shell-doping in GaSb/InAs core-shell nanowires with various diameters. The behaviors of Zn doping the shell of GaSb/InAs nanowires with different diameters and core sizes were also investigated, as shown in Fig. 4. The GaSb/InAs diameters approximately vary from 2.5 to 3.5 nm, the corresponding GaSb-core diameters increase from 1.5 to 2.0 nm, and the InAs-shell thicknesses change from 1.0 to 1.5 nm. The stable substituting sites for Zn substituting the shell In atoms were selected to locate at the interface based on the above discussion. The band structures and charge density distributions of Zn shell doping GaSb/InAs core shell nanowires were given in Fig. 4. It is observed that the acceptor level is higher for Zn doping the core-shell with a small GaSb-core size as compared with doping the large core sizes, as shown in the band structures of Fig. 4(a). On the other hand, as the core-shell nanowires with a fixed GaSb-core diameter, the acceptor level is also slightly higher for Zn doping the core-shell nanowires with a thicker InAs-shell, as can be seen in the Fig. 4(a). From the charge density distributions of acceptors states, we observe the hole charge density mainly localized at GaSb-core. The hole state originated from Zn-doped InAs-shell is transferred into non-doped GaSb, realizing remote *p*-type doping.

It should be emphasized that remote *p*-type doping can be successfully achieved in bare ZB [111] GaSb/InAs core-shell NW without being affected by surface states, while that cannot be realized in bare ZB [110] core-shell NW. It could be inferred that the orientation of side facet of NWs greatly influence the *p*-type doping behavior. To estimate the side facets stabilities, we calculated the surface energies of InAs (111), (110), and (100) surface slabs according to the InAs-shell side facets in the GaSb/InAs NWs as shown in Fig. 1(a). The surface slabs were modeled using periodic slabs with ten bilayers separated by 15 Å to ensure a convergence of the surface energy and atomic relaxations. It is found that surface energies of InAs (111), (110), and (100) surface slabs are 0.117 eV/ Å^2^, 0.055 eV/ Å^2^, and 0.158 eV/Å^2^, respectively, namely, *E*_surf_ (100) > *E*_surf_ (111) > *E*_surf_ (110). The InAs (110) surface is found to be energetically more favorable than the InAs (111) and (100) surface slabs due to the lower density of dangling bonds.

**Figure 5:**
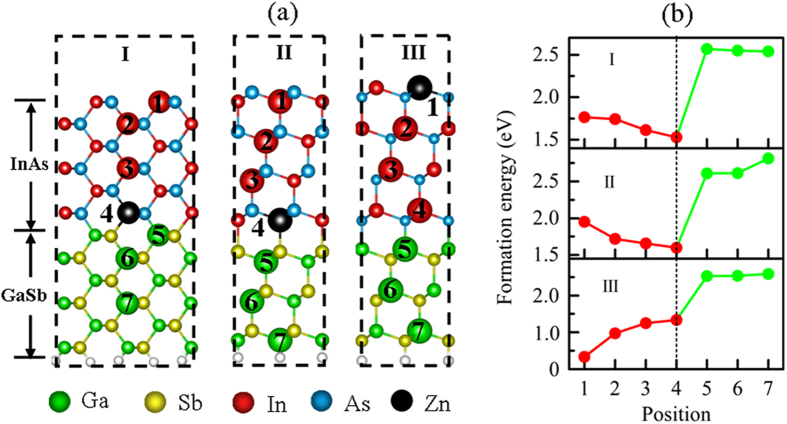
(**a**) Side viewed of (2 × 2) GaSb/InAs two-dimensional heterogeneous slabs with (110) In-As atomic pairs terminated surface (I-panel), (111) As atoms terminated surface (II-panel), and (111) In atoms terminated surface (III-panel), (**b**) corresponding formation energy as a function of different Zn substituting positions shown in (**a**). The (110) and (111) heterogeneous slabs consist of 12 and 14 atomic layers, respectively. Green, yellow, red, blue balls and black represent Ga, Sb, In, As, and Zn atoms, respectively. The bottoms of heterogeneous slabs were GaSb atoms passivated by pseudo-hydrogen atoms (white spheres).

In order to understand the effects of side facets of NWs on the electronic properties of remote *p*-type doping in core-shell NWs, we explore the behavior of Zn doping in the GaSb/InAs heterogeneous slabs with different surface planes. The positions of Zn substituting are marked in Fig. 5(a). The corresponding substituting formation energies are given in Fig. 5(b). We can find from Fig. 5(b) that, whatever the slabs terminations are, the formation energies of doping in InAs shell are lower than that of doping in GaSb core. It suggests that Zn doping in the InAs is more energetically favorable than in the GaSb. For doping in the (110) In-As atomic pairs terminated slab, Zn preferably substitutes the interfacial In atom in the site 4 due to the lowest formation energies shown in I-panel of Fig. 5(b). It agrees well with the case of Zn preferably substitutes the interfacial In atom of bare [111] GaSb/InAs core-shell NW. For doping in (111) As-terminated slab, the formation energies of doping in the interfacial In atom in the site 4 is also the lowest than that of other substituting positions as seen in II-panel of Fig. 5(b). This can be understood from a coupling between the Zn impurity acceptor and surface acceptor states. Due to the unsaturated dangling bonds of surface As atoms, the acceptors states are formed at the slab surface. A Coulomb repulsion between the surface acceptors states and the Zn impurity acceptor state has taken place. The strong coupling drives the Zn atom from surface to the interface, decreasing the energy of system. However, in the case of doping (111) In-terminated slabs, as shown in III-panel of Fig. 5(b), the formation energies of Zn substituting the surface In atom (site 1) is the lowest than that of substituting other positions. The Zn impurity is trapped at the surface of slab. It is because that surface donors states originating from unsaturated dangling bonds of surface In atoms has a Coulomb attraction with the Zn impurity acceptor state. So the most energetically favorable for Zn is to substitute In atoms in the site 1 of the surface plane.

**Figure 6:**
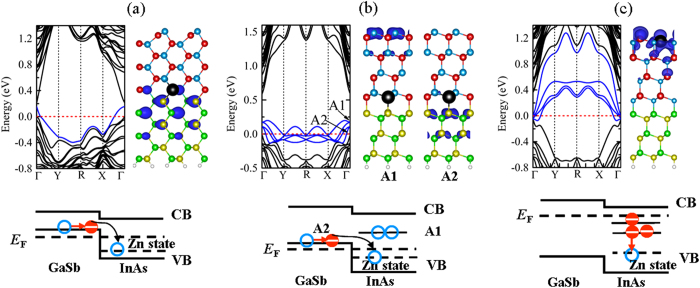
Band structure (left panel) and charge density distributions (right panel) of Zn substituting: (**a**) the In atom in the site 4 of (110) In-As atomic pairs terminated, (**b**) the In atom in the site 4 of (111) As atoms terminated, and (**c**) the surface In atom in the site 1 of (111) In atoms terminated GaSb/InAs heterogeneous slabs, respectively. The corresponding doping mechanism schematic models are displayed in the lower panel. The charge density distributions in (**a**) and (**b**) correspond to the acceptor states, and that in (**c**) correspond to the donor states. The blue lines in the band structures of (**a**) and (**b**) represent the acceptors levels, and that of (**c**) represent the donor levels, respectively. The isosurface value of charge density is at the 30% of the maximum.

We focus on studying the electronic properties of Zn substituting the stable doping positions of the heterogeneous slabs. The corresponding band structures and charge density distributions are shown in Fig. 6. From Fig. 6(a), we find the band structure shows a *p*-type characteristic when Zn substitute the interfacial In atom of (110) heterogeneous slab. The charge density distribution of acceptor state reveals that the acceptor state wave function mainly locates in the GaSb region. The doping mechanism shown in the lower panel of Fig. 6(a) is the same as the case of Zn doing the [111] GaSb/InAs core-shell NW. A remote *p*-type doping has been realized in 2D (110) GaSb/InAs heterogeneous slab. For the case of substituting the (111) heterogeneous slab with As atoms termination, as can be seen in Fig. 6(b), it is found that two double-degenerate acceptors (labeled by A1 and A2 states, respectively) are created, crossing the Fermi level. The A1 state is higher in energy than the A2 state. From the distribution of the acceptors states charge wave function, we observe that the charge density distribution of A2 acceptor state localizes at the GaSb-region, and that of A1 acceptor state localizes at the surface As atoms which indicates that the A1 states mainly originate from the unsaturated dangling bonds of surface As atoms. The doping mechanism is a little different from that doping in (110) heterogeneous slab as seen in the lower panel of Fig. 6(b). The energy level of A1 acceptor state is higher than that of GaSb valence band, whereas the energy level of GaSb valence band is higher than that of Zn impurity state. Due to the band offset at the GaSb/InAs interface, the electrons of GaSb are spontaneously transferred into the Zn impurity acceptor state, leading to the creation of hole state A2 at the GaSb core NW. So one-dimensional hole gas is formed, and localized at the GaSb atoms. The *p*-type doped InAs contribute hole to the non-doped GaSb. However, for the case of doping in (111) heterogeneous slab as shown in Fig. 6(c), the band structure presents metallic characteristic. The surface donor states originating the unsaturated dangling bonds of surface In atoms are created below the InAs conduction bands. The doping mechanism is schematically sketched in the lower panel of Fig. 6(c). We can see that the surface donor states could directly compensate the Zn impurity acceptor state. One-dimensional hole gas cannot be survival in the slab system with In-atom terminated surface plane, resulting low hole concentration. In general, we conclude that the remote *p*-type doping can be successfully realized in GaSb/InAs heterogeneous (110) slabs and (111) As-atom terminated slab. From the behavior of doping in the 2D slabs, we infers that remote *p*-type doping could be achieved in the GaSb/InAs core-shell NWs that with (110) side facets and (111) side facets with As atoms termination, such as ZB [001] and wurtzite (WZ) [0001] GaSb/InAs core-shell NWs.

## Discussion

In summary, we have performed the first-principles calculations to study the formation energies and electronic properties structures of Zn *p*-type doing in GaSb/InAs core-shell NWs and GaSb/InAs heterogeneous slabs. It is found that Zn atom preferably doping in the interfacial In atoms of InAs-shell in bare ZB [111] GaSb/InAs core-shell NW. Because of the band offset at the [111] GaSb/InAs core-shell NW interface, the *p*-doped InAs shell spontaneously donates free holes to the non-doped GaSb core without the need of thermal activation, realizing remote *p*-type doping and increasing the hole mobility and concentration of GaSb NW. The remote *p*-type doping in InAs shell separate hole gas from the impurity atoms, decreasing the impurity scattering. The bare ZB [110] GaSb/InAs core-shell NW is not suitable for *p*-type doping due to the compensation from the surface donors states. In order to examine the effect of side facets of NWs on remote *p*-type doping, we studied the Zn doping properties of 2D GaSb/InAs heterogeneous slabs with three types of surface atoms termination. The results show that Zn doping in the InAs is more stable than in the GaSb regions. For the GaSb/InAs heterogeneous slabs oriented along (110) surface and (111) surface slabs with As atoms termination, Zn doped-InAs contribute hole carriers to the GaSb without being compensated by surface free electron. From the behaviors of doping in the slabs, we infers that remote *p*-type doping can be achieved in the GaSb/InAs core-shell NWs that with (110) side facets and (111) side facets with As atoms termination, such as ZB [001] and WZ [0001] GaSb/InAs core-shell NWs. These results might be useful for designing high-mobility and fast speed nanoelectronic devices.

## Method

Our calculations were performed using the Vienna ab initio Simulation Package (VASP) based on the density functional theory (DFT)^38^,^39^. The exchange-correlation energy was described in the generalized-gradient approximation (GGA) potential^40^. The energy cutoff for the plane-wave expansion was set to 370 eV using project-augmented wave (PAW) potential^41^. The bare ZB GaSb/InAs core-shell NWs were constructed along the [111] and [110] directions as shown in Fig. 1(a), respectively. The dangling bonds at the corner of [111] core-shell NW were removed, since the corner effect of nanowires on the transport properties were very weak at large diameters nanowires, and the side facets effect on the electronic properties were mainly discussed in our study. All the atoms of core-shell NWs and heterogeneous slab were optimized until the total energies converged to below 10^−4^ eV and the forces acting on atoms were less than 10^−2^ eV/ Å. The Monkhorst-Pack grids of k-point mesh were set 1 × 1 × 7 for GaSb/InAs core-shell NWs and 5 × 5 × 1 for GaSb/InAs heterogeneous slabs^42^. A vacuum region of greater than 10 Å was created in the directions perpendicular to NW and to slabs axis, respectively. The formation energies of Zn substituting In (Zn_In_) atoms of InAs-shell and Ga (Zn_Ga_) atoms of GaSb-core were calculated based on the formulation described as^43^:
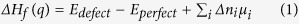
Where 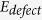 and 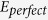 are the total energies of core-shell NWs with and without the Zn doped-atom, respectively. 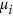 is the chemical potential of constituent 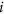 in the As-rich regime for Zn_In_ in the InAs-shell and in the Sb-rich regime for Zn_Ga_ in the GaSb-core, respectively. 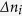 is the difference between the number of atomic species 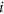 in the Zn-doped and non-doped GaSb/InAs NWs. For one-dimensional (1D) materials, carrier mobility can be estimated by deformation potential theory under the effective mass approximation and the electron acoustic-phonon scattering mechanism^32^,^33^,^34^,^35^,^36^,^37^.
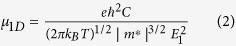
in which 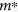 is the carrier effective mass, 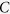 is the stretching modulus of one-dimensional system, 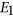 is the deformation potential constant of band edge, and the *T* is the temperature taken as 300 K. Here, the effective mass can be calculated by 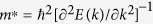, where 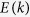 is the energy band. The stretching modulus of one-dimensional system is defined as 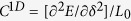, where the uniaxial strain 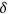 is applied along the nanowires grown direction, and 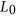 is the periodic length of periodic unite cell of core-shell NWs along the *z*-direction. The deformation potential constant is proportional to the stretching-induced band-edge shift caused by external strain, 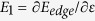, where 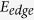 is the energy level of CBM or VBM. The surface energies can be calculated according to^44^:
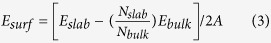
Where 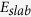 and 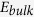 are the energies of the surface slab and bulk, respectively. 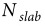 and 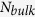 are the number of atoms pairs in the 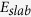 and 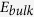, respectively. 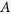 is the surface area of the surface slab.

## Additional Information

**How to cite this article**: Ning, F. *et al.* Remote *p*-type Doping in GaSb/InAs Core-shell Nanowires. *Sci. Rep.* **5**, 10813; doi: 10.1038/srep10813 (2015).

## Figures and Tables

**Table 1 t1:** Stretching modulus (*C*), electron effective mass ( 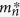 ), hole effective mass ( 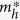 ), deformation potential constants of conduction band edge (*E*_1(CBE)_) and valence band edge (*E*_1(VBE)_) and carriers mobilities of electron ( 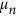 )and hole ( 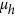 ) of pristine non-doped GaSb/InAs core-shell NW and Zn doping in the shell of ZB [111] GaSb/InAs core-shell.

	***C***	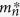	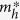	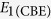	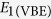	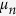	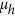
**Core-shell NWs**	**(eV/Å)**	( 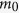 )	( 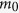 )	**(eV)**	**(eV)**	**(10**^**3**^**cm/Vs)**	**(10**^**3**^**cm/Vs)**
Non-doped	469.6806	0.0721	0.0944	8.83844	5.34315	2.45	4.48
Zn doping the shell	460.6061	0.0725	0.0835	8.7425	5.22446	2.44	5.53
